# Predicting positron emission tomography brain amyloid positivity using interpretable machine learning models with wearable sensor data and lifestyle factors

**DOI:** 10.1186/s13195-023-01363-x

**Published:** 2023-12-12

**Authors:** Noriyuki Kimura, Tomoki Aota, Yasuhiro Aso, Kenichi Yabuuchi, Kotaro Sasaki, Teruaki Masuda, Atsuko Eguchi, Yoshitaka Maeda, Ken Aoshima, Etsuro Matsubara

**Affiliations:** 1 Department of Neurology, Faculty of Medicine, Oita University, Idaigaoka 1-1, Hasama, Yufu, Oita 879-5593 Japan; 2 Microbes & Host Defense Domain Deep Human Biology Learning, Eisai Co., Ltd, 5-1-3, Tokodai, Tsukuba-Shi, Ibaraki 300-2635 Japan; 3 School of Integrative and Global Majors, University of Tsukuba, Tennoudai 1-1-1, Tsukuba, Ibaraki 305-8577 Japan

**Keywords:** Amyloid positivity, Lifestyle factors, Machine learning, Mild cognitive impairment, Wearable sensor, PiB-PET

## Abstract

**Background:**

Developing a screening method for identifying individuals at higher risk of elevated brain amyloid burden is important to reduce costs and burden to patients in clinical trials on Alzheimer’s disease or the clinical setting. We developed machine learning models using objectively measured lifestyle factors to predict elevated brain amyloid burden on positron emission tomography.

**Methods:**

Our prospective cohort study of non-demented, community-dwelling older adults aged ≥ 65 years was conducted from August 2015 to September 2019 in Usuki, Oita Prefecture, Japan. One hundred and twenty-two individuals with mild cognitive impairment or subjective memory complaints (54 men and 68 women, median age: 75.50 years) wore wearable sensors and completed self-reported questionnaires, cognitive test, and positron emission tomography imaging at baseline. Moreover, 99 individuals in the second year and 61 individuals in the third year were followed up. In total, 282 eligible records with valid wearable sensors, cognitive test results, and amyloid imaging and data on demographic characteristics, living environments, and health behaviors were used in the machine learning models. Amyloid positivity was defined as a standardized uptake value ratio of ≥ 1.4. Models were constructed using kernel support vector machine, Elastic Net, and logistic regression for predicting amyloid positivity. The mean score among 10 times fivefold cross-validation repeats was utilized for evaluation.

**Results:**

In Elastic Net, the mean area under the receiver operating characteristic curve of the model using objectively measured lifestyle factors alone was 0.70, whereas that of the models using wearable sensors in combination with demographic characteristics and health and life environment questionnaires was 0.79. Moreover, 22 variables were common to all machine learning models.

**Conclusion:**

Our machine learning models are useful for predicting elevated brain amyloid burden using readily-available and noninvasive variables without the need to visit a hospital.

**Trial registration:**

This prospective study was conducted in accordance with the Declaration of Helsinki and was approved by the local ethics committee of Oita University Hospital (UMIN000017442). A written informed consent was obtained from all participants. This research was performed based on the Strengthening the Reporting of Observational Studies in Epidemiology reporting guideline.

## Background

Dementia is a growing public health issue in an aging society with an increasing life expectancy that poses a serious social and economic impact in patients and caregivers. Alzheimer’s disease (AD) is a major cause of dementia in people aged over 65 years. Recently, an anti-amyloid β (Aβ) antibody has been approved by the Food and Drug Administration as a new disease-modifying therapy for AD. This agent reduced brain amyloid deposition and the rate of cognitive decline in the mild cognitive impairment (MCI) or mild dementia stage [1]. Therefore, more precise and early identification of patients with AD is crucial to enhance the benefit from disease-modifying therapy. The neuropathologic hallmarks of AD are the extracellular aggregation of Aβ plaque, the presence of neurofibrillary tangles, and neuronal degeneration [2, 3]. Aβ accumulation in the brain is a key pathological characteristic and the initial event in the pathological process of AD [4, 5]. Therefore, Aβ is the major target of disease-modifying therapy in clinical trials on AD [6, 7]. However, almost all agents were not effective due to delayed intervention or selection of inappropriate participants without amyloid pathology [7, 8]. ^11^C-Pittsburgh Compound B (PiB) positron emission tomography (PET) and measurement of cerebrospinal fluid (CSF) Aβ levels are established biomarkers of amyloid pathology, and they can predict the incidence of AD before the onset of dementia [2, 3]. Nevertheless, their use in clinical and research setting are limited due to their high cost or the need for invasive lumbar puncture. Moreover, the low prevalence of amyloid positive MCI in multicenter research (46.6%) or population-based study (22%) may cause higher cost and burden to the patients due to a large number of PiB-PET scan or CSF analysis in clinical trials on AD or clinical setting after the availability of disease modifying therapy [9, 10]. Therefore, it is challenging to develop a cost-effective and noninvasive method for detecting amyloid pathology prior to PiB-PET or CSF analysis. Although blood-based biomarkers are a promising solution for predicting individuals with amyloid pathology in the memory clinics [11–14], they are not suitable for population screening due to the need to visit a hospital and draw blood. In this study, we developed and validated predictive models using three machine learning techniques by integrating easily available and noninvasive lifestyle variables to detect individuals with elevated brain amyloid deposition. Moreover, overall daily physical activity and sleep patterns was objectively and continuously collected using wearable sensors without recall bias [15, 16]. These predictive models can be useful for the population-wide screening or primary care setting to identify the patients eligible for PiB-PET or CSF analysis. Almost all previous studies have developed machine learning models for classifying individuals with healthy cognition, MCI, and AD, or for predicting the incidence of dementia [17, 18]. Meanwhile, a few studies have applied machine learning techniques in neuropsychological tests, neuroimaging, and blood-based biomarker analysis combined with demographic characteristics and APOE genotype for predicting brain amyloid positivity [19–26]. To the best of our knowledge, few studies have focused on lifestyle factors as predictive variables for identifying individuals at high risk for brain amyloid deposition [27]. There is growing evidence obtained from cohort studies showing that physical inactivity, social isolation, sleep disturbance, depression, and vascular risk factors are important predictors of late-life cognitive impairment [28–30]. Similarly, our prospective cohort study showed an association between objectively measured modifiable lifestyle factors using wearable sensor with cognitive function, brain amyloid deposition, or cortical glucose metabolism in community-dwelling older adults [31, 32]. These results lead us to the hypothesis that machine learning models using objectively measured lifestyle factors may predict elevated brain amyloid deposition measured via PiB-PET. Therefore, the current study aimed to predict brain amyloid positivity with three machine learning models using wearable sensor data alone or in combination with demographic characteristics and living environment and health behaviors. Our models are different form previous machine learning models as they used lifestyle factors, which can be assessed using wearable sensors and questionnaires without visiting a hospital. Further, this study developed and validated predictive models using three machine learning techniques by integrating easily available and noninvasive lifestyle variables to detect individuals with elevated brain amyloid deposition. These models can be implemented widely for the population-based prescreening to detect amyloid positivity and can reduce unnecessary invasive lumbar puncture and PET scans, leading to successful clinical trials on AD and maximize the therapeutic effect of disease-modifying therapy.

## Materials and methods

### Participants

The USUKI study was prospective in nature and was conducted on community-dwelling older adults without dementia from August 2015 to September 2019 in Usuki, Oita Prefecture, Japan. It was designed to explore the risk and protective lifestyle factors of cognitive decline in later life. Detailed designs and methods have been described elsewhere [31, 32]. The inclusion criteria were as follows: 1) age ≥ 65 year, 2) living in Usuki, 3) physically and psychologically healthy, 4) absence of dementia, and 5) independent function in activities of daily living. All participants were required to wear a wristband sensor for 7 consecutive days every 3 months (four times per year) over 3 years of follow-up. The valid sensing data were defined as at least 3 days in one period and at least two period in a year. Table 1 shows the clinical and demographic characteristics of the participants. Between August 2015 and October 2017, 855 older individuals (317 men [37.1%] and 538 women [62.9%], median age [interquartile range (IQR)]: 73 [69–78] years, median education duration [IQR]: 12 (11–12) years) satisfied the criteria, and they had valid sensing data for analysis at baseline (data not shown). Of 855 individuals, 122 (54 men [44.3%] and 68 women [55.7%], median age [IQR]: 75.50 [71.00–80.00] years, median education duration [IQR]: 12 [9–12] years) with MCI or subjective memory complaints (118 presented with MCI and 4 with subjective memory complaints) who underwent cognitive test and PiB-PET at baseline were recruited in the current study. The diagnosis of MCI was made based on the criteria of previous studies, which were as follows: 1) subjective and objective memory impairment, 2) Clinical Dementia Rating score of 0.5, and 3) absence of significant impairment in cognitive function or activities of daily living. Data on demographic characteristics and living environment and health behaviors were collected by trained medical staff using self-reported questionnaires (clinical history and medication). Moreover, the cognitive test and PiB-PET were conducted on 99 individuals in the second year and 61 individuals in the third year (Table 1). In total, 282 eligible records with valid wearable sensor, cognitive test, and PiB-PET findings and data on demographic characteristics and living environment and health behavior were used in the machine learning models.

**Table 1:** Clinical and demographic characteristics of the participants

Characteristics of the participants^a^	First year (*n* = 122)	Second year (*n* = 99)	Third year (*n* = 61)
**Demographic characteristics**			
Age, years, median (IQR)	75.50 (71.00–80.00)	76.00 (72.00–80.00)	76.00 (71.00–80.00)
Sex (male:female)	54:68	44:55	29:32
Education duration, median (IQR)	12.00 (9.00–12.00)	12.00 (9.00–12.00)	12.00 (9.00–12.00)
BMI, kg/m^2^, median (IQR)	23.19 (21.28–24.93)	23.46 (21.66–25.37)	23.60 (22.00–25.32)
ApoE4 (negative:positive)^b^	105:17	85:14	49:12
Ever drinking (none:sometimes:everyday)	78:23:21	62:20:17	40:14:7
Ever smoking (none:sometimes:everyday)	117:0:5	95:1:3	60:0:1
**Wearable sensor data**			
Steps/day, median (IQR)	4425.21 (2839.88–6364.50)	4168.86 (2552.55–5886.65)	4008.35 (2542.67–5420.50)
Light physical activity, min/day, median (IQR)	16.46 (10.55–28.64)	18.79 (10.07–29.06)	16.05 (8.71–23.21)
Moderate-to-vigorous physical activity, min/day, median (IQR)	21.46 (11.16–35.13)	17.82 (10.76–29.95)	15.30 (10.46–29.83)
Sedentary behavior, min/day, median (IQR)	788.35 (739.65–830.27)	779.83 (724.03–839.19)	776.26 (713.50–842.21)
TST, min/day, median (IQR)	400.12 (355.96–445.53)	401.85 (357.71–455.97)	412.75 (371.41–474.67)
Sleep efficiency/day, median (IQR)	0.96 (0.93–0.97)	0.96 (0.94–0.98)	0.96 (0.94–0.97)
Awakening time count, times/day, median (IQR)	0.47 (0.29–0.72)	0.43 (0.29–0.68)	0.47 (0.25–0.68)
Naptime, min/day, median (IQR)	35.50 (19.23–59.73)	37.77 (23.43–63.95)	47.27 (25.71–68.78)
Nap efficiency/day, median (IQR)	0.99 (0.93–1.00)	0.98 (0.95–1.00)	0.96 (0.94–1.00)
Awakening time count during nap, times/day, median (IQR)	0.03 (0.00–0.10)	0.03 (0.00–0.08)	0.04 (0.02–0.08)
Heart rate, beats/min, median (IQR)	63.48 (59.63–68.79)	63.05 (59.00–69.20)	62.83 (58.97–69.88)
Conversation time, min/day, median (IQR)	213.79 (173.46–270.91)	221.59 (183.01–279.64)	221.72 (176.75–271.07)
ActiveScale, times/day, median (IQR)	4.63 (3.18–6.70)	4.59 (2.87–6.80)	3.84 (2.46–5.91)
**Others**			
MoCA-J score, median (IQR)	22.00 (19.00–25.00)	23.00 (20.00–26.00)	23.00 (19.00–25.00)
Mean SUVR on PiB, median (IQR)	0.92 (0.83–1.32)	0.90 (0.83–1.37)	0.90 (0.82–1.49)
PiB-PET (negative:positive)	94:28	75:24	45:16

*IQR* Interquartile range

*BMI* Body mass index, *min* minute, *TST* Total sleep time

^a^All variables, except ApoE4, were used before the missing value was imputed

^b^The value in the 2nd and 3rd years of ApoE4 was imputed using the values in the 1st year

### Demographic characteristics of the participants

Data on demographic characteristics, such as age, sex, education duration, and body mass index (BMI), were collected by trained medical staff annually. Moreover, a history of chronic disease, such as hypertension, diabetes mellitus, hyperlipidemia, stroke, heart disease, liver dysfunction, renal dysfunction, thyroid disease, and malignant tumor, was assessed based on clinical history and medications used.

### Living environment and health behaviors

Different living environment and health behaviors (Table 2) were collected using self-report questionnaires annually. We focused on family structure, living conditions (with a relative), transportation, engagement in paid work, hobby, exercise habits, cognitive activity, and social relationship. Dichotomous variables such as drug and food allergy, pet ownership, gardening engagement, and cohabitants were used. Categorical variables such as ever smoking or drinking (none, sometimes, everyday), history of chronic diseases (none, previous treatment, and current treatment), walking difficulty (none, walking with pain, need to use a cane), transportation mode (bicycle, driving one’s self in a private car or motorcycle, train or bus, and riding with family or friends or taxis), accompanying person (a person who can accompany the participants to outpatients visit: relative, friend, and others), caring about appearance (not at all, not often, very often, and most of the time), and denture (use of denture: none, partial denture, and full denture) were used. The four- or five-point frequency scale was used for the number of outing (none, 1–2 days a week, 3–4 days a week, and ≥ 5 days a week), reading a newspaper (none, 1–2 days a month, 1–2 days a week, 3–4 days a week, and ≥ 5 days a week), time spent on watching TV (none, < 3 h per day, > 3 h per day, and > 6 h per day), and lesson or class frequency (the frequency of taking lesson or class: none, 1–2 days a month, 1–2 days a week, 3–4 day a week, ≥ 5 days a week), communication frequency (the frequency of communication with friends or relatives: none, 1–2 days a month, 1–2 days a week, 3–4 a week, and ≥ 5 days a week), and primary or secondary hobby (none, 1–2 days a month, 1–2 days a week, 3–4 day a week, and ≥ 5 days a week). The number of family living together represents household size, and the number of days working in a week (the average number of days spent on paid work), exercise frequency, and the number of days participating in community activity were treated as continuous variables.

**Table 2:** Explanatory variables with summary statistics in this study

**Characteristics of patients**^**a**^	**Amyloid negative (*n* = 214)**	**Amyloid positive (*n* = 68)**	***P*-value**^b^
**Demographic characteristics (17 variables)**			
Age, years, median (IQR)	75.00 (70.00–79.00)	79.00 (76.00–82.25)	< 0.01
Sex (male:female)	105:109	22:46	0.02
Education duration, median (IQR)	12.00 (9.00–12.00)	12.00 (12.00–12.00)	0.08
BMI, kg/m^2^, median (IQR)	23.59 (21.53–25.32)	22.91 (20.73–24.79)	0.22
Ever drinking (none:sometimes:everyday)	129:47:38	51:10:7	0.03
Ever smoking (none:sometimes:everyday)	205:0:9	67:1:0	0.28
Hypertension (none:previous treatment:current treatment)	95:7:112	19:5:44	0.04
Diabetes mellitus (none:previous treatment:current treatment)	164:4:46	55:4:9	0.37
Hyperlipidemia (none:previous treatment:current treatment)	152:7:55	42:7:19	0.25
Stroke (none:previous treatment:current treatment)	206:4:4	57:9:2	< 0.01
Heart disease (none:previous treatment:current treatment)	186:9:19	47:9:12	< 0.01
Liver dysfunction (none:previous treatment:current treatment)	207:3:4	66:1:1	0.89
Renal dysfunction (none:previous treatment:current treatment)	211:3:0	68:0:0	0.33
Thyroid disease (none:previous treatment:current treatment)	204:5:5	55:9:4	< 0.01
Malignant tumor (none:previous treatment:current treatment)	198:14:2	63:5:0	0.96
Medicine allergies (no:yes)	206:8	66:2	0.76
Food allergies (no:yes)	203:11	63:5	0.49
**Wearable sensor data (13 variables)**			
Steps/day, median (IQR)	4492.77 (2923.02–6207.15)	2944.68 (1579.57–5448.91)	< 0.01
Light physical activity, min/day, median (IQR)	17.24 (10.70–27.88)	14.02 (7.83–30.86)	0.79
Moderate-to-vigorous physical activity, min/day, median (IQR)	20.23 (12.38–33.69)	10.77 (5.07–27.26)	< 0.01
Sedentary behavior, min/day, median (IQR)	779.39 (717.21–830.35)	808.79 (754.28–846.99)	< 0.01
TST, min/day, median (IQR)	405.32 (362.22–457.00)	389.47 (356.20–434.32)	0.03
Sleep efficiency/day, median (IQR)	0.96 (0.94–0.98)	0.95 (0.94–0.97)	0.09
Awakening time count, times/day, median (IQR)	0.46 (0.26–0.68)	0.45 (0.31–0.73)	0.34
Naptime, min/day, median (IQR)	40.01 (22.22–68.85)	36.32 (23.97–53.72)	0.55
Nap efficiency/day, median (IQR)	0.98 (0.94–1.00)	0.97 (0.94–1.00)	0.29
Awakening time count during nap, times/day, median (IQR)	0.03 (0.00–0.09)	0.03 (0.00–0.08)	0.44
Heart rate, beats/min, median (IQR)	64.06 (59.66–69.77)	61.12 (56.18–65.70)	< 0.01
Conversation time, min/day, median (IQR)	212.68 (173.30–264.98)	258.27 (202.89–304.63)	< 0.01
ActiveScale, times/day, median (IQR)	4.88 (3.34–6.77)	3.00 (1.68–5.81)	< 0.01
**Health and life environment questionnaire data (23 variables)**			
Household size, number of people, median (IQR)	2.00 (2.00–3.00)	2.00 (2.00–3.00)	0.57
Living with spouse (no:yes)	58:156	17:49	0.83
Living with children (no:yes)	133:81	46:20	0.27
Living with grandchildren (no:yes)	179:35	55:11	0.95
Living with parents (no:yes)	206:8	64:2	0.79
Living with others (no:yes)	210:4	65:1	0.85
Transportation mode (bicycle:driving a car or motorcycle:train or bus:family/friends’ cars or taxis)	11:142:12:44	0:25:5:35	< 0.01
Accompanying person (relative:friend:others)	162:2:49	63:0:1	< 0.01
Number of days working in a week, median (IQR)	0.00 (0.00–0.00)	0.00 (0.00–0.00)	0.08
Primary hobby (none:1–2 days a month:1–2 days a week:3–4 days a week:5 days or more a week)	65:31:34:22:40	30:4:9:3:13	0.14
Secondary hobby (none:1–2 days a month:1–2 days a week:3–4 days a week:5 days or more a week)	53:57:30:24:13	20:16:13:5:7	0.98
Gardening engagement (no:yes)	158:56	55:13	0.24
Walking difficulty (none:walking pain:cane)	154:25:15	42:7:9	0.20
Number of outings (none:1–2 days a week:3–4 days a week:5 days or more a week)	6:40:71:90	3:20:21:21	0.04
Exercise frequency, days, median (IQR)	4.00 (1.50–7.00)	3.00 (2.00–7.00)	0.83
Communication frequency (none:1–2 days a month:1–2 days a week:3–4 days a week:5 days or more a week)	37:85:44:27:15	6:11:24:13:11	< 0.01
Pet ownership (no:yes)	145:64	51:15	0.22
Lesson or class frequency (none:1–2 days a month:1–2 days a week:3–4 days a week:5 days or more a week)	158:35:7:4:2	47:4:10:2:1	0.33
Caring about appearance (not at all:not often:very often:most of the time)	5:61:70:74	1:15:26:24	0.47
Denture (none:partial:yes)	69:109:33	19:31:14	0.37
Time spent on watching TV (none: < 3 h per day: > 3 h per day: > 6 h per day)	7:73:104:29	1:22:31:12	0.44
Reading a newspaper (none:1–2 days a month:1–2 days a week:3–4 days a week:5 days or more a week)	26:9:11:14:150	3:3:2:4:54	0.07
Number of days participating in community activity, median (IQR)	0.00 (0.00–1.00)	0.00 (0.00–1.00)	0.85
**Cognitive test**			
MoCA-J score, median (IQR)	23.00 (20.00–25.75)	20.00 (18.00–22.00)	< 0.01

*IQR* Interquartile range

*BMI* Body mass index, *min* minute, *TST* Total sleep time

^a^Each variable was used after the missing value imputed using same ID's value

^b^Welch two-sample two-sided t-test; Wilcoxon rank sum test

### Wearable sensor data

All participants wore a wristband sensor (Silmee™ W20, TDK Corporation, Tokyo, Japan) continuously except when bathing. Physical activity, sleep, conversation time, and heart rate were calculated by summing up the sensor data captured on each day and by averaging the entire measurement period annually. Data that indicated removing the wristband sensor according to heart rate were excluded. Physical activity data were detected using a three-axis accelerometer that measured acceleration in three perpendicular axes. The steps and intensity of activity as metabolic equivalents (METs) were captured. Physical activity intensity was divided into three categories, which were as follows: sedentary behavior (≤ 1.5 METs), light physical activity (LPA) (1.6–2.9 METs), and moderate-to-vigorous physical activity (MVPA) (≥ 3.0 METs) [33]. The period of sedentary behavior, LPA, and MVPA was evaluated during awaking. Sleep–wake variables such as total sleep time (TST), sleep efficiency, time awake after sleep onset (WASO), and awakening time count, were assessed from 18:00 to 5:59 the following day. Sleep onset was defined as the first 20-min block of resting state without movement. Nocturnal waking and waking during nap were defined as 5–90 min of continuous movement during a continuous sleep period. Sleep efficiency was calculated as the rate of TST versus the time spent in bed. WASO was defined as the total number of minutes awake after sleep onset during the night. Daytime napping was defined as the resting period without movement on the wearable sensor from 6:00 in the morning to 17:59 in the evening. Nap efficiency was calculated as rate of naptime versus the time spent resting during daytime. Notably, WASO was not used as variable in machine learning in this study. Heart rate was calculated by obtaining the average pulses per minute in each day. Moreover, our wearable sensor could detect sound pressure levels for utterances that originated within a 2-m radius from the device. The sound pressure level ranged from 55 to 75 dBA at this distance. Sound data were continuously captured via a microphone on the wearable sensor and were analyzed to evaluate conversation time. The microphone on the sensor could not detect the content of conversations. ActiveScale was calculated by counting the number of hours spent on walking at least 250 steps.

### Cognitive function

Cognitive assessments were performed using the Japanese version of the Montreal Cognitive Assessment (MoCA-J), with a score of 0–30. MoCA-J comprises seven subscales, which are as follows: 1) visuospatial/executive function (alternate trail making, cube copying, and clock-drawing task, 5 points), 2) naming (three animal figures, 3 points), 3) memory (repetition only, no point), 4) attention (forward and backward digit span, target detection using tapping, and serial 7 subtraction, 6 points), 5) language (repetition, and verbal fluency, 3 points), 6) abstraction (2 points), 7) memory (enumeration of 5 nouns after approximately 5 min, 5 points), and 8) orientation (time and place, 6 points) [34]. If the education duration was ≤ 12 years, we added one point to the total score of MoCA-J according to previous studies.

### Apolipoprotein E phenotype

The ε4 allele of the apolipoprotein E gene is a genetic risk factor for late-onset AD and is associated with brain amyloid deposition [35]. The apolipoprotein E gene could not be conducted. Hence, the apolipoprotein E (APOE) phenotype was examined. The APOE phenotype was identified using the Human Apolipoprotein E4/Pan-APOE ELISA kit (MBL Co., Ltd., Woburn, the USA), which measures the amount of APOE4 or total APOE specifically with high sensitivity using affinity-purified polyclonal antibody against APOE and monoclonal antibody against APOE4 using sandwich enzyme-linked immunosorbent assay. Moreover, it assesses differences among the homozygotes (i.e., ε4/ε4) and heterozygotes (ε2/ε4, ε3/ε4) of the APOE4 phenotypes and non-APOE4 zygotes (ε2/ε2, ε3/ε3, and ε2/ε3) based on the APOE level-to-APOE4 level ratio. The homozygotes or heterozygotes of the ApoE4 phenotypes, and non-ApoE4 zygotes were defined based on a cutoff of 0.3 [36]. Notably, the ApoE4 phenotype was not used as variable in machine learning in this study.

### Positron emission tomography scans

Siemens Biograph mCT (Siemens) in the three-dimensional scanning mode was used in static 11C-PiB-PET studies. The production of PET tracers was performed based on good manufacturing standard at the PET Center of Oita University Hospital. Further, 11C -PiB (mean: 547 MBq [SD: 60]) was injected intravenously as a rapid bolus with a saline flush, and radioactivity concentrations were measured from 50 to 70 min after injection. Radiation in pre- and post-dose samples was measured to define the exact injected dose using a radiation detector. All imaging data were reconstructed using the following parameters: thick slice: 3.0-mm, matrix: 256 × 256, and magnification: 3.0 × . The pixel size of the reconstructed images was 1.06 mm. Spatial normalization of PiB scans was performed with a customized PET template at the Montreal Neurological Institute reference space using the Statistical Parametric Mapping version 8 (Wellcome Trust Centre for Neuroimaging). The region of interest (ROI), including the frontal lobes, temporoparietal lobes, posterior cingulate gyrus, and cerebellum, was determined using the MarsBaR (MRC Cognition and Brain Sciences Unit) ROI toolbox for Statistical Parametric Mapping, as described in a previous study [32]. These ROIs included areas with known amyloid deposition in patients with AD [37]. The average ROI values was obtained across both hemispheres. The standardized uptake value ratio (SUVR) was calculated from the voxel number-weighted average of the median uptake in the frontal, temporoparietal, and posterior cingulate ROIs relative to the ROI in the cerebellum [38]. The single mean value of all regions was combined to represent the global SUVR for PiB-PET. Amyloid positivity was defined as a global PiB SUVR of ≥ 1.4. Meanwhile, amyloid negativity was defined as a global PiB SUVR of < 1.4 for machine learning models.

### Statistical analysis

Continuous and categorical variables were summarized as medians with IQR and ratios, respectively in Tables 1 and 2. To assess the distribution balance of explanatory variables between the amyloid positive and negative groups, we used Welch’s t-test for continuous variables and Wilcoxon rank-sum test for categorical variables. All statistical analyses were conducted using Python version 3.7.7.

### Model construction and evaluation

Models were constructed based on machine learning algorithms for predicting brain amyloid deposition. Three steps were conducted in model construction, i.e., Step 1: pre-selecting the machine learning algorithms, Step 2: feature selection, and Step 3: training and evaluating the models. Step 1: To select the proper machine learning algorithms, DataRobot 7.1.3 (DataRobot, the USA) was employed to explore different types of machine learning algorithms. We found that three algorithms (i.e., kernel Support Vector Machine [SVM], Elastic Net, and logistic regression) had a higher accuracy after investigating more than 2500 machine learning algorithms. SVM is supervised learning algorithms which is less affected by outlier, kernel SVM can be applied to nonlinear classification by incorporating kernel functions to algorithm. In this study, we used the radial basis function kernel (RBF kernel) as the kernel function in the SVM. Elastic Net is linear regression algorithm combined L1 and L2 regularization methods. Logistic regression is classification algorithms. In this study we set L2 penalty in logistic regression as regularizer. Step 2: To establish a predictive model for amyloid positivity using accumulated data collected using wearable sensor and questionnaires, the selection of proper number of variables is the key point to prevent overfitting in the machine learning algorithm. We pre-selected 54 variables as the predictors from the cognitive test score variables and 111 variables including demographic characteristics (35), wearable sensor (24), and living environment and health behaviors (52) before training the models. In the pre-selection procedure, as a first step, a comprehensive correlation calculation was conducted among 111 variables to avoid including statistically similar variables in the models. One of two variables whose correlation coefficient equals 1.0 were excluded from the candidate predictive variables. Second, variables that were related to outcome in a clinical point of view were selected from the candidate variables that remained after the first step. Table 2 shows summary statistics of all predictive variables in the machine learning algorithm. The variables with missing values were imputed using only training data before training. Thereafter, we explored different types of machine learning models using 52 variables that excluded MoCA-J score and ActiveScale data from 54 variables for predicting PET amyloid positivity (Model 0–3). The reason why MoCA-J score was excluded from the variables set was to validate the performance of models using variables that do not require a visit to the hospital. And the reason for the exclusion of ActiveScale was to compare the performances of models using ActiveScale with ones not using it and examine whether we could use ActiveScale as a substitute for other exercising features, which is easy to be calculated from questionnaires and hence effective for future social implementation. Step 3: For training and validation of the machine learning models, hyper-parameter tuning was conducted using nested cross-validation, the hyper-parameters in algorithms were chosen from some candidate values using grid-search method within training dataset, and the exact grid of parameters in each algorithm were, gamma: [0.0001, 0.001, 0.01] and C: [1, 10, 100] in kernel SVM, L1_ratio: [0.01, 0.1, 0.9] and C: [0.1, 1, 10] in Elastic Net, C: [0.01, 0.03, 0.1, 0.3, 1, 3, 10, 30, 100] in logistic regression, respectively. All variables were preprocessed with standardization or smooth rigid transform depending on the types of variables. In model construction, the Boruta method was employed as the feature selection technique for reducing variables that did not contribute to the discrimination of amyloid positivity from the amyloid negative group and improvement of explanation ability [39]. Using Boruta method, features were selected in the training models. Feature selection with Boruta was also conducted within the cross-validation loop using only training data in this study. The other machine learning models were also constructed by combining variables after feature selection using the Boruta method with MoCA-J scores or ActiveScale data (Model 4–6). In order to avoid information leakage of test dataset and biased evaluation of model performance, we used the group fivefold cross validation to assess our models, in which records from the same participant were assigned to the same fold. No holdout was set in training the models. In this study, each model was trained with fivefold cross validation with 10 different seeds (10 repeats), and mean of area under a receiver operating characteristic curve (ROC AUC) was utilized for evaluation. Also, other evaluation indices, precision, recall, and F1 score as a harmonic mean of precision and recall, were calculated. In this study, the cutoff point to calculate these indices was selected using Youden index computed from training dataset. The ROC curve for the fivefold cross validation in the fifth constructed models from the top of 10 times seeds randomization were also outputted to confirm generalized performance. In addition, the permutation importance of each variable finally remained in each model was computed as feature importance [40]. The series of steps in model training and performance validation were conducted using Python version 3.7.7.

## Results

### Clinical and demographic characteristics of the participants

Table 1 shows data on the demographic characteristics, wearable sensor parameters, cognitive function, and PiB uptake value of the participants. At baseline, the median age (IQR) of the participants was 75.50 (71.00–80.00) years, and 68 (55.7%) were women. The median education duration (IQR) was 12 (9–12) years, and the median BMI (IQR) was 23.19 (21.28–24.93). In terms of physical activity variables, the median daily steps (IQR) was 4425.21 (2839.88–6364.50), and the median times spent on LPA, MVPA, and sedentary behavior (IQR) were 16.46 (10.55–28.64), 21.46 (11.16–35.13), and 788.35 (739.65–830.27) min/day, respectively. In terms of sleep variables, the median daily TST (IQR) was 400.12 (355.96–445.53) min, the median daily sleep efficiency (IQR) was 0.96 (0.93–0.97). The median daily conversation time (IQR) was 213.79 (173.46–270.91) min, and the median daily heart rate (IQR) was 63.48 (59.63–68.79) beats/day. The walking steps and sleep parameters of our participants were similar to those in previous studies that assessed Japanese adults of a similar age [41, 42]. The median MoCA-J score (IQR) was 22 (19–25). The median PiB SUVR (IQR) was 0.92 (0.83–1.32). Based on a PiB-PET SUVR cutoff of 1.4, 28 (23.0%) of 122 individuals were amyloid positive. In addition, 17 (13.9%) individuals were APOE4 carriers. Therefore, the prevalence of individuals with abnormal PiB uptake was relatively small than that of individuals in previous studies [10, 43]. Of individuals with MCI followed up to the second year (*N* = 34) and the third year (*N* = 61), 1 and 2 respectively converted to dementia in the second and the third years, while 3 and 17 reverted to normal cognition. The average annual conversion and reversion rate were 1.9% and 12.8%, respectively.

### Model prediction accuracy

**Fig. 1:**
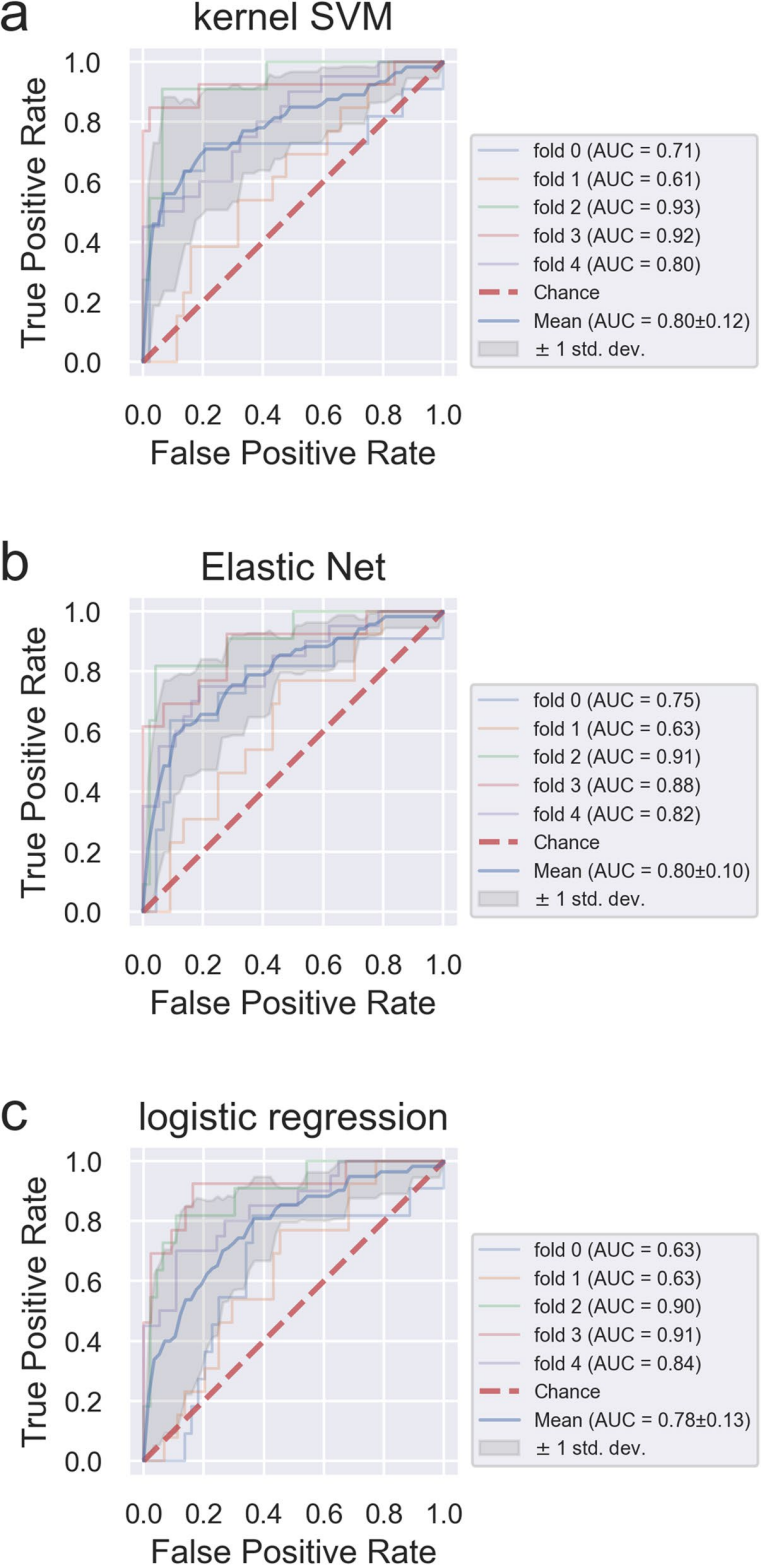
Receiver operating characteristic (ROC) curves in each three machine learning models. Every ROC curve represents the results in the fifth model from the top of 10 times seeds for the fivefold cross validation results of predicting amyloid positivity. The blue line shows the mean ROC for the fivefold cross validation, and the red dot line shows the chance and ROC of each fivefold according to different colors. The gray shadow shows ± 1 standard deviation of the mean ROC. **a** kernel SVM, **b** Elastic Net, **c** logistic regression

**Fig. 2:**
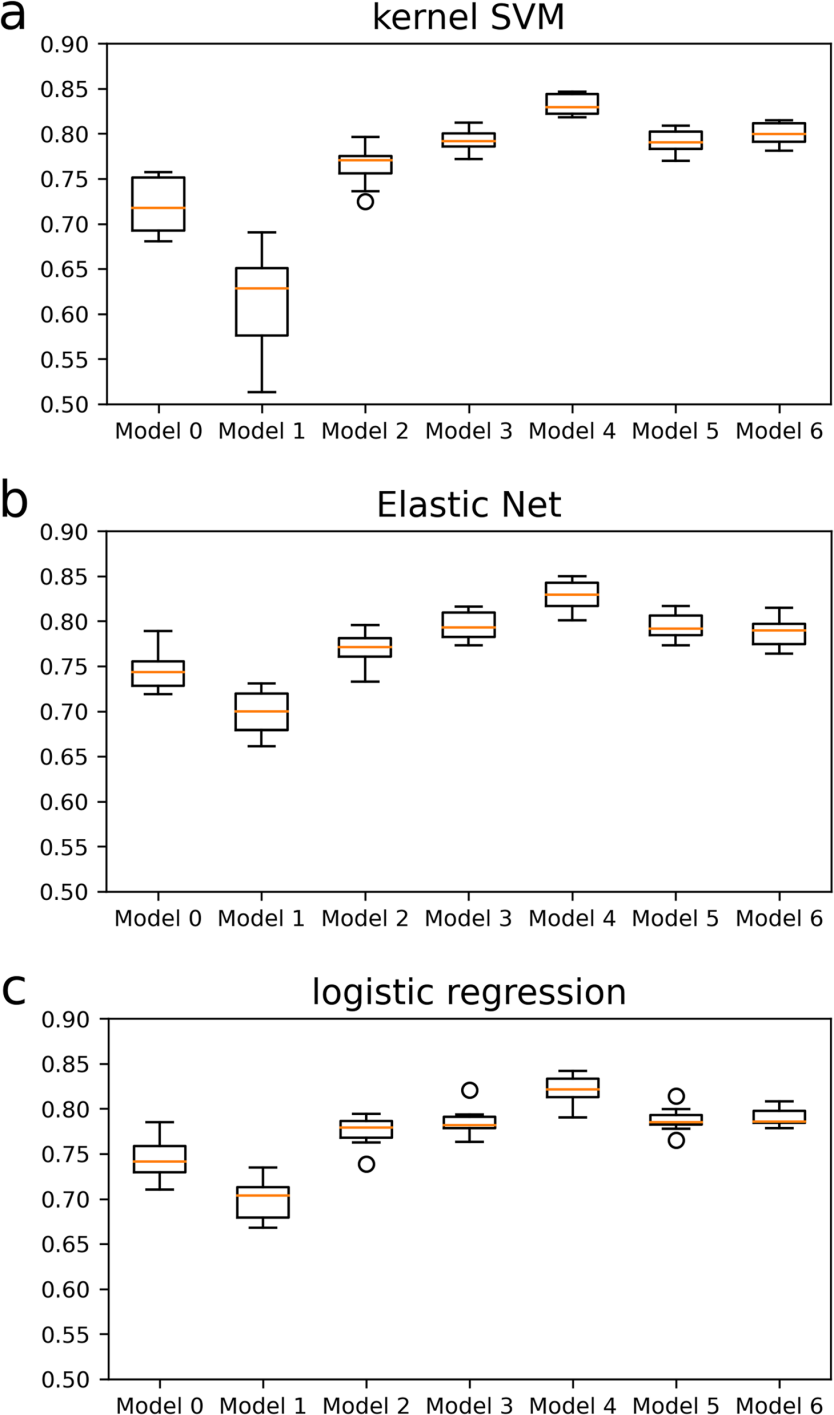
The boxplot of the cross-validated AUCs across all 10 repeats. The flier points are those past the end of the whiskers extending from the box by 1.5 × the inter-quartile range (IQR). **a** kernel SVM, **b** Elastic Net, **c** logistic regression

The current study applied machine learning models for assessed data on 54 variables collected from 282 records, which included valid wearable sensor, cognitive test, and PiB-PET imaging data. Among 282 records, 68 (24.1%) and 214 (75.9%) indicated amyloid positivity and negativity (Table 2). Three machine learning models (namely, kernel SVM, Elastic Net, and logistic regression) were constructed using 25–37 variables (25 for kernel SVM, 35 for Elastic Net, and 37 for logistic regression). These variables were selected from initial 54 variables, except two variables of MoCA-J score and ActiveScale using the feature selection algorithm Boruta method. These variables were defined as basic features. The other machine learning models were also constructed using basic features combined with MoCA-J scores or ActiveScale data. The machine learning models were constructed not only by adding basic features, but also by replacing exercise variables of basic features because ActiveScale information is a substituted variable for exercise habits. In addition, to confirm the contribution of variables in basic features, the machine learning models were also established using age, sex, education years and BMI (Model 0 in Table 3), only wearable sensor variables or wearable sensor and demographic variables with feature selection using the Boruta method (Model 1–6 in Table 3). Table 3 shows the mean predictive accuracy of each model and combination dataset. The mean ROC AUC of 10 times seeds were 0.79 for all three models, using basic features (wearable sensor, demographic characteristics, health and life environment questionnaire features) (Model 3 in Table 3). Precision in Model 3 were 0.49, 0.51, 0.51 for kernel SVM, Elastic Net, and logistic regression, and regarding recall, the values showed 0.69, 0.63, 0.58 for kernel SVM, Elastic Net, and logistic regression, respectively. The F1 score, a harmonic mean of precision and recall, were 0.56, 0.55, 0.51 for kernel SVM, Elastic Net, and logistic regression, respectively. Furthermore, MoCA-J was combined separately with basic features and used to construct the three models. The predictive accuracy improved from 0.79 to 0.83 after combining basic features with the total MoCA-J score (Model 4 in Table 3). Precision and recall of three algorithms in Model 4 went up or down comparing with Model3, and the F1 score (Model 4 in Table 3) was slightly higher than before combining the total MoCA-J score (Model 3 in Table 3) in all three algorithms. Moreover, the ROC AUC of the models with basic features and ActiveScale data was approximately 0.80, which was similar to that of Model 3, and the ROC AUC was similar even after removing exercising features including steps, period of sedentary behavior, LPA, and MVPA (Models 5, 6 in Table 3). About Model 5 and Model 6, other evaluation indices except ROC AUC showed similar tendency of Model 3 and Model 4. The mean ROC AUC of 10 times seeds were 0.61, 0.70, 0.70 for kernel SVM, Elastic Net, and logistic regression, respectively, using wearable sensor variables alone (Model 1 in Table 3). However, the values of precision, recall, F1 score showed considerably low than the values of Model 3 in all algorithms. The ROC AUC of the models with wearable sensor and demographic variables had fair performance (approximately 0.77, Model 2 in Table 3). As for precision, recall and F1 score of these models, not as much as them of Model 1, showed low values. In addition, the mean ROC AUC were 0.72, 0.75, 0.74, precision were 0.41, 0.42, 0.43, recall were 0.63, 0.71, 0.69, and F1 score were 0.48, 0.52, 0.51 for kernel SVM, Elastic Net, and logistic regression, respectively (Model 0 in Table 3). Figure 1 shows the ROC curves of each machine learning model using basic features (Model 3 in Table 3) showed the variation of the fifth constructed models from the top of 10 times seeds randomization for the fivefold cross validation. The mean AUC as the mean cross-validation performance of our models, which had generalized performance, were 0.80 ± 0.12 SD for kernel SVM, 0.80 ± 0.10 SD for Elastic Net, and 0.78 ± 0.13 SD for logistic regression. Figure 2 shows the boxplot of the cross-validated AUCs across all 10 repeats in each model, Model 3 and Model 4 demonstrated higher performance than either Model 0 using age, sex, education years and BMI or Model 1 using wearable sensor data only.

**Table 3:** Evaluation indices of amyloid positivity prediction models using machine learning methods

	**Model type**	**ROC AUC**	**Precision**^**h**^	**Recall**^**h**^	**F1 Score**^**h**^
**Model 0**^**a**^	kernel SVM	0.72 ± 0.03	0.41 ± 0.03	0.63 ± 0.08	0.48 ± 0.04
	Elastic Net	0.75 ± 0.02	0.42 ± 0.02	0.71 ± 0.06	0.52 ± 0.04
	L2 Logistic Regression	0.74 ± 0.02	0.43 ± 0.02	0.69 ± 0.06	0.51 ± 0.04
**Model 1**^**b**^	kernel SVM	0.61 ± 0.06	0.30 ± 0.04	0.48 ± 0.08	0.37 ± 0.04
	Elastic Net	0.70 ± 0.02	0.39 ± 0.02	0.62 ± 0.04	0.47 ± 0.02
	logistic regression	0.70 ± 0.02	0.41 ± 0.02	0.60 ± 0.05	0.47 ± 0.04
**Model 2**^**c**^	kernel SVM	0.76 ± 0.02	0.45 ± 0.01	0.66 ± 0.05	0.52 ± 0.02
	Elastic Net	0.77 ± 0.02	0.44 ± 0.03	0.71 ± 0.06	0.51 ± 0.04
	logistic regression	0.78 ± 0.02	0.45 ± 0.03	0.67 ± 0.06	0.51 ± 0.03
**Model 3**^**d**^	kernel SVM	0.79 ± 0.01	0.49 ± 0.04	0.69 ± 0.05	0.56 ± 0.03
	Elastic Net	0.79 ± 0.01	0.51 ± 0.05	0.63 ± 0.06	0.55 ± 0.05
	logistic regression	0.79 ± 0.01	0.51 ± 0.03	0.58 ± 0.05	0.51 ± 0.04
**Model 4**^**e**^	kernel SVM	0.83 ± 0.01	0.56 ± 0.03	0.67 ± 0.04	0.60 ± 0.03
	Elastic Net	0.83 ± 0.02	0.56 ± 0.03	0.64 ± 0.05	0.58 ± 0.03
	logistic regression	0.82 ± 0.02	0.55 ± 0.02	0.61 ± 0.04	0.55 ± 0.02
**Model 5**^**f**^	kernel SVM	0.79 ± 0.01	0.49 ± 0.03	0.67 ± 0.03	0.55 ± 0.03
	Elastic Net	0.79 ± 0.01	0.51 ± 0.04	0.64 ± 0.04	0.54 ± 0.04
	logistic regression	0.79 ± 0.01	0.52 ± 0.04	0.60 ± 0.04	0.54 ± 0.03
**Model 6**^**g**^	kernel SVM	0.80 ± 0.01	0.51 ± 0.03	0.67 ± 0.04	0.56 ± 0.02
	Elastic Net	0.79 ± 0.02	0.53 ± 0.04	0.62 ± 0.04	0.53 ± 0.03
	logistic regression	0.79 ± 0.01	0.53 ± 0.04	0.58 ± 0.04	0.53 ± 0.03

^a^Model 0: Age, Sex, Education duration, BMI

^b^Model 1: Wearable sensor data with feature reduction

^c^Model 2: Wearable sensor data, demographic characteristics with feature reduction

^d^Model 3: Wearable sensor data, demographic characteristics, health and life environment questionnaire data with feature reduction

^e^Model 4: Wearable sensor data, demographic characteristics, health and life environment questionnaire data with feature reduction + MoCA-J score

^f^Model 5: Wearable sensor data, demographic characteristics, health and life environment questionnaire data with feature reduction + ActiveScale

^g^Model 6: Wearable sensor data, demographic characteristics, health and life environment questionnaire data with feature reduction + ActiveScale (as a substitute for exercising features)

^h^Cutoff point was selected using the Youden index

### Visualization of feature importance

**Fig. 3:**
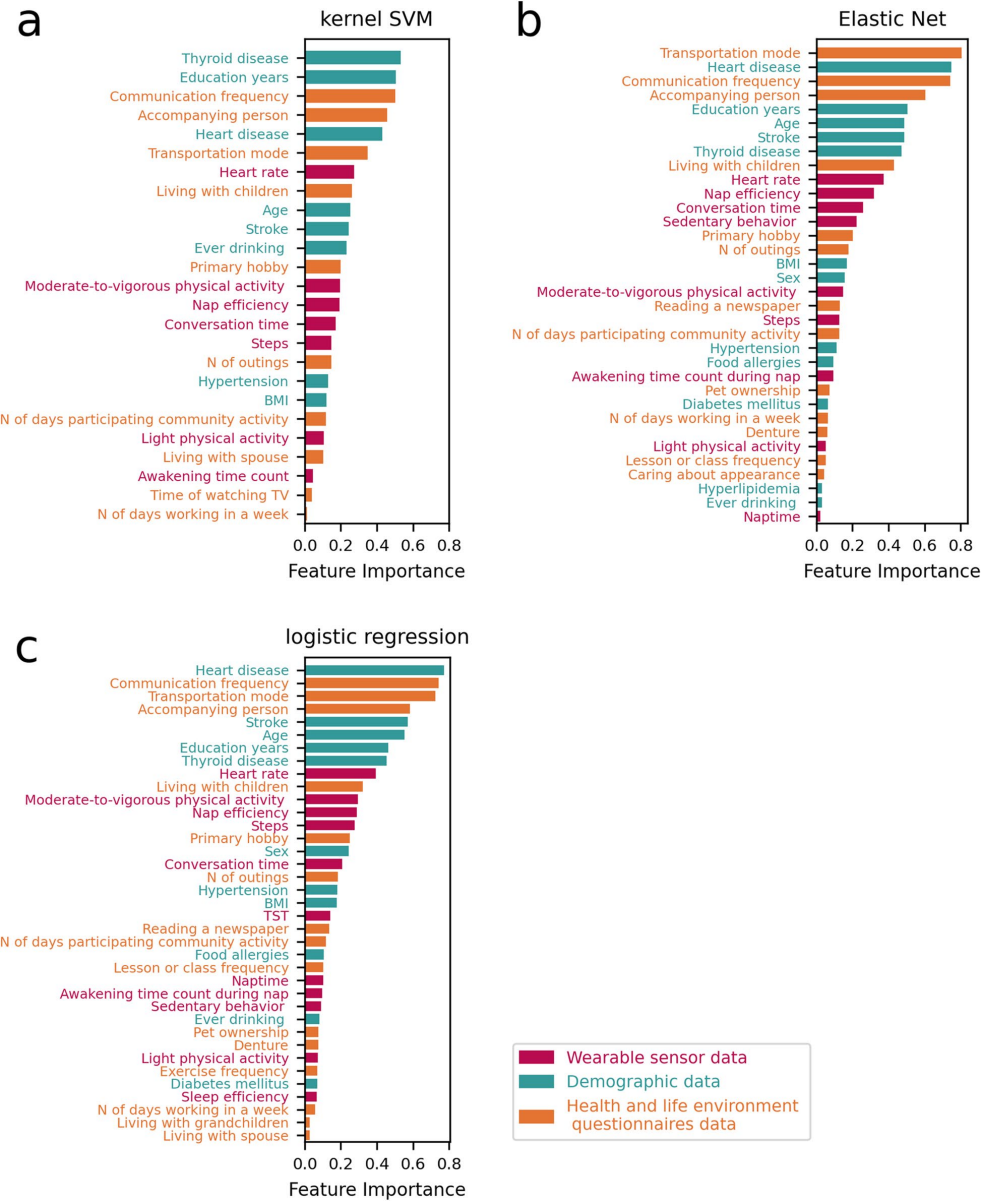
The feature importance ranking table extracted in each three machine learning models. The vertical axis labels show the explanatory variables, and the horizontal axis labels depict the feature importance of each explanatory variable. **a** kernel SVM, **b** Elastic Net, **c** logistic regression

**Fig. 4:**
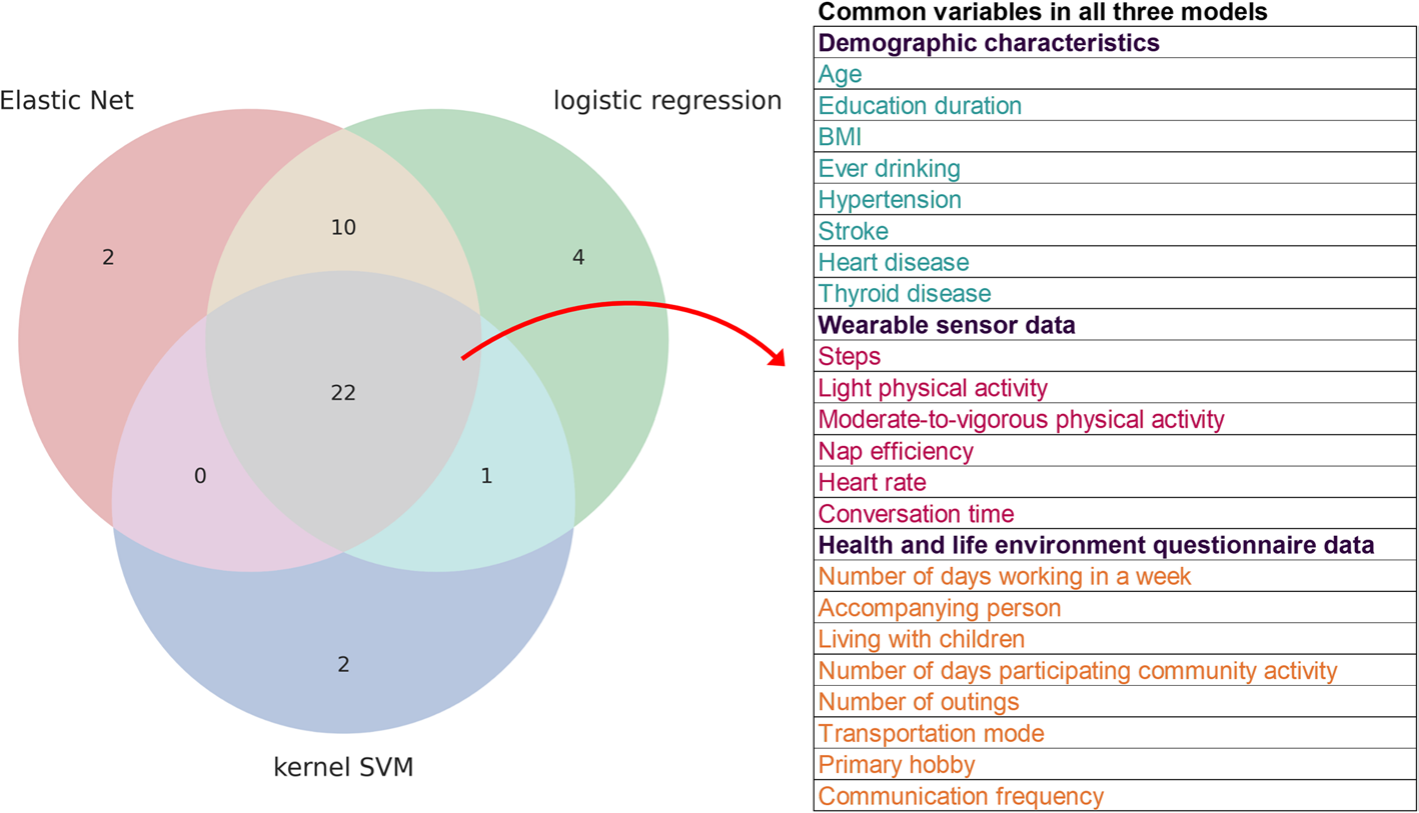
Variables that remained in each of the model. Venn diagram showing the variables that finally remained among the three models and 22 common variables in all three models

To make the machine learning interpretable and explainable, the feature importance of all models were performed using the permutation importance estimate method. Figure 3 shows the feature importance listing for features. In total, 25 features were extracted in the kernel SVM, which included the following: 8 features related demographic characteristics and chronic diseases (age, education duration, BMI, ever drinking, hypertension, stroke, heart disease, and thyroid disease); 3 features related to physical activities (steps, LPA, and MVPA), 2 features related to sleep parameters (nap efficiency and awakening time count), heart rate, and conversation time from wearable sensor; 10 features related to living environment and health behaviors (living with spouse or children, transportation mode, accompanying person, number of days working in a week, primary hobby, time spent on watching TV, number of days participating in community activity, communication frequency, and number of outings). Similarly, 34 features were extracted in the Elastic Net model, which included the following: 12 features related to demographic characteristics and chronic diseases (age, sex, education duration, BMI, ever drinking, food allergies, hypertension, diabetes mellitus, hyperlipidemia, stroke, heart disease, and thyroid disease); 4 features related to physical activities (steps, LPA, MVPA, and sedentary behavior), 3 features related to sleep parameters (naptime, nap efficiency, and awakening time count during nap), heart rate, and conversation time from wearable sensor; 13 features related to living environment and health behaviors (living with children, transportation mode, accompanying person, number of days working in a week, primary hobby, reading a newspaper, lesson or class frequency, number of days participating in community activity, communication frequency, number of outings, pet ownership, caring about appearance, and denture). Third, 37 features were extracted in the logistic regression model, which included the following: 11 features related to demographic characteristics and chronic diseases (age, sex, education duration, BMI, ever drinking, food allergies, hypertension, diabetes mellitus, stroke, heart disease, and thyroid disease); 4 features related to physical activities (steps, LPA, MVPA, and sedentary behavior), 5 features related to sleep parameters (TST, sleep efficiency, naptime, nap efficiency, and awakening time count during nap), heart rate, and conversation time from wearable sensor data; 15 features related to living environment and health behaviors (living with spouse, children, or grandchildren, transportation mode, accompanying person, number of days working in a week, primary hobby, exercise frequency, reading a newspaper, lesson or class frequency, number of days participating in community activity, communication frequency, number of outings, pet ownership, and denture). Finally, as shown in Fig. 4, 22 variables were common to all three machine learning models: 8 features were related to demographic characteristics and chronic diseases (such as age, education duration, BMI, ever drinking, hypertension, stroke, heart disease, and thyroid disease), 3 features related to physical activities (such as steps, LPA, and MVPA), 1 feature related to sleep parameter (such as nap efficiency), heart rate, and conversation time, and 8 features related to living environment and health behaviors (such as living with children, transportation mode, accompanying person, number of days working in a week, primary hobby, number of days participating in community activity, communication frequency, and number of outings).

## Discussion

The current study established machine learning models for predicting PET amyloid positivity using three categories (objectively measured lifestyle factors alone or in combination with demographic characteristics and living environment and health behaviors). The machine learning technique was used to evaluate 54 variables collected from 122 participants without dementia at baseline (118 with MCI and 4 with subjective memory complaints). Further, the models integrating three categories had a better predictive accuracy (AUC of 0.79) than the models using wearable sensor data alone (AUC of 0.70). Moreover, the predictive accuracy (AUC of 0.83) slightly improved with the integration of MoCA-J with three categories. Given the performance of previous studies and the aim of our study model to be utilized for prescreening of amyloid positivity in the brain, we considered the performance in this study (ROC AUC = 0.79) to be fair and acceptable in real-world settings. To the best of our knowledge, this study first developed and validated models using machine learning techniques to predict PET amyloid positivity with lifestyle variables collected using wearable sensors and questionnaires in community-dwelling older adults. These machine learning models are useful for prescreening to detect amyloid positivity and can reduce costs and the number of unnecessary invasive lumbar puncture and amyloid PET in the clinical trials on AD or clinical settings. The most interesting finding of the current study was that machine learning models using objectively measured lifestyle factors combined with demographic characteristics and living environment and health behaviors had acceptable performance on predicting amyloid positivity on PET. These models, which are based on different machine learning techniques, had similar predictability (0.79 in all three algorithms). This finding suggested that the ability of each model is reliable for learning and making associations within and between data despite the utilization of different classification techniques. Research on predictive models have investigated several machine learning techniques. The different machine learning techniques have the similar predictive performance for amyloid positivity and negativity. This finding increases the reliability and generalizability of predictive models [44]. However, the AUC value slightly increased if the MoCA-J score was added (kernel SVM: 0.83, Elastic Net: 0.83, and logistic regression: 0.82 respectively). The assessment of MoCA-J required the trained medical staffs or clinical psychologists. Several studies have developed machine learning models to predict brain amyloid deposition using demographic characteristics, neuropsychological test results, APOE genotype, neuroimaging findings, and blood-base biomarkers. Some studies have reported the use of two predictive models using demographic characteristics and neuropsychological test results. The AUC of the model using age, family history, online cognitive function instrument scores, and Cogstate was 0.806 [19]. The AUC of the model that used Mini-Mental State Examination (MMSE) score, Alzheimer’s Disease Assessment Scale, American National Adult Reading Test, Rey Auditory Verbal Learning Test, clock drawing, and logical memory delayed recall was 0.864 [20]. Moreover, four models have used the APOE genotype combined with demographic characteristics and neuropsychological test. The AUC values were 0.65 in the model using age, sex, education duration, ApoE4, baseline cognition, and longitudinal cognitive rate [21], 0.72 in the model using age, sex, education duration, ApoE4, and neuropsychological tests (such as MMSE score, Alzheimer’s disease assessment scale, and logical memory II) [22], and 0.83 in the model using age, 10-word delayed recall, and ApoE4 [23]. The model using age, sex, education duration, history of hypertension, ApoE4, and word list recall test score had the highest accuracy at 0.873 [24]. Machine learning models using neuroimaging or blood-base biomarkers combined with demographic characteristics, ApoE4, and neuropsychological testes had better performance in predicting brain amyloid positivity. The AUC of the model using MRI radiomic features combined with age, sex, and ApoE4 was 0.79 [25]. The AUC of the model using MRI volumetrics combined with age, sex, education duration, ApoE4, and neuropsychological tests was 0.71 [22]. The AUC of the model using six blood-based markers combined with age, ApoE4, and CDR was 0.87 [11]. The AUC of the model using plasma Aβ42/Aβ40 combined with age, 10-word delayed recall score, and ApoE4 was 0.85 [23]. The AUC of the model using blood derived p-tau or amyloid beta in a series of studies was exceeding 0.80 [12, 13]. There was the study reporting the capacity of plasma Aβ42/Aβ40 measured using six different assays can predict amyloid positivity with good performance [14]. Another study also showed that good amyloid PET positivity prediction accuracy by using MMSE, age, and APOE in addition to blood test [26]. These findings suggested that machine learning models combined with demographic characteristics, cognitive test results, APOE genotype, neuroimaging, or blood-base biomarkers can be used to identify individuals with elevated brain amyloid deposition. The model prediction accuracy of the current study was comparable to that in previous studies. Note that some of the previous studies listed above differ with our study in that they used study population including cognitively normal participants [11–13, 19–21]. Moreover, there was a study establishing the models using lifestyle factors and other dominant variables such as APOE, MMSE, and in its study, although study population is participants including cognitively normal, the model using only lifestyle factors was also established [27]. The advantage of our models is that lifestyle factors objectively measured with wearable sensor and living environment and health behaviors are completely noninvasive and easily available in the community.

The three machine learning techniques used common variables, such as demographic characteristics, chronic diseases, physical activity, nap, heart rate, conversation time, and living environment and health behaviors in the predictive models. These variables are important for differentiating individuals who are positive for amyloid from those who are negative for amyloid. The important predictors included demographic characteristics such as age, education duration, BMI, ever drinking, hypertension, stroke, heart disease, and thyroid disease. Most of the previous machine learning models for predicting brain amyloid deposition included age, sex, and education duration. It is well established that advanced age is the greatest risk factor for AD [45] and associated with the higher prevalence of Aβ positivity [46, 47]. The proportion of individuals with normal cognition who are positive for amyloid generally increased with aging [10, 47]. Education is considered as an indicator of cognitive reserve and individuals with higher level of education have greater brain amyloid burden than those with lower levels of education [48–50]. Sex did not remain as common variables in all three models. Although the incident rate of AD is higher in women than man [45], the association between sex and brain amyloid burden is controversial [51, 52]. BMI may be bi-directionally associated with brain amyloid burden. Is has been reported that greater brain amyloid burden was associated with the subsequent decline in BMI and higher BMI was associated with greater brain amyloid burden [53, 54]. Moderate alcohol intake was associated with a lower risk of cognitive impairment or brain amyloid deposition in older adults [55, 56]. Moreover, a growing body of evidence has shown the association between vascular risk factors, including hypertension, and hyperlipidemia or cerebrovascular disease, and AD pathology [57, 58]. By contrast, the association between diabetes mellitus and brain amyloid deposition is inconsistent [59, 60]. The two-hit vascular hypothesis of AD has proposed that vascular risk factors contribute to the dysregulation of neurovascular unit, thereby resulting in chronic hypoperfusion or impaired Aβ clearance and increased Aβ production [58]. The association between cardiovascular disease and AD pathology remains unclear [61]. However, several autopsy cases showed that coronary artery disease was associated with brain amyloid deposition [62]. Thyroid state was associated with brain Aβ deposition [63, 64]. In particular, triiodothyronine negatively regulates the gene expression of amyloid precursor protein [64, 65]. The important predictors in wearable sensor data included physical activity, nap efficiency, heart rate, and conversation time. Physical activity or exercise is associated with lower brain amyloid deposition on PET and higher Aβ42 levels in the cerebrospinal fluid among older adults without dementia [66]. The mechanisms underlying the association between physical activity and brain amyloid deposition suggests that physical activity inhibits amyloid production and enhances amyloid degradation or clearance [67, 68]. Although short sleep duration, poor sleep quality, and frequent napping are associated with higher brain amyloid deposition [28], only few studies have investigated the role of nap efficiency. Previous findings on the association between daytime napping and cognitive function have been contrasting. Self-reported daytime napping reduced the risk of cognitive decline [69]. Meanwhile, more frequent napping measured using actigraphy was associated with a poorer cognitive function [70]. Further studies should be conducted to validate the association between nap efficiency and brain amyloid deposition. A higher resting heart rate is a risk factor for not only stroke or cardiovascular disease but also cognitive decline or dementia in older adults [71]. Nevertheless, the association between heart rate and brain amyloid deposition remains unclear. The important predictors related to living environment and health behaviors included living together with a relative, transportation mode, number of days working in a week, hobby, exercise frequency, and social relationship. Conversation time, living with children, number of days participating in community activity, communication frequency, and number of outings are related to social isolation or loneliness. Older adults with less social participation and contact and subjective loneliness are at higher risk for cognitive impairment and dementia [72]. Moreover, active social engagement, including contact with family and friends and positive social support and engagement in leisure activities, play a role in preventing cognitive impairment [73]. The association between loneliness and brain amyloid deposition has been found in older adults with healthy cognition [74]. The transportation mode and accompanying person (the need for company during hospital visits) were important predictive variables in each model. The number of older adults who retire from driving increased according to stringent licensing polices in Japan. Older adults who stop driving are at high risk of depression, general health decline, cognitive impairment, social isolation, and mortality [75, 76]. Although alternative transportation is required to maintain independent mobility for shopping or social connectedness, the public transport network is inadequate particularly in rural areas. A higher level of AD biomarkers in the CSF could be a determiner of early driving cessation among older adults [77]. Moreover, transportation with family or friend is attributed to impairment in instrumental activities of daily living, which is associated with brain amyloid deposition [78]. Number of days working in a week is a protective factor against decline in cognitive function or basic activities of daily living. However, it is not a predictor of elevated brain amyloid deposition in a longitudinal observational cohort study [79]. This finding is inconsistent with that of our machine learning models probably due to differences in study design, analytic methods, or age of participants.

The current study had several strengths. That is, lifestyle factors, such as physical activity, sleep, and conversation, were continuously and accurately measured using a wearable sensor in community-dwelling older adults. Further, brain amyloid deposition was assessed via PiB-PET.

### Limitations

The current study had several limitations that should be considered. First, the predictive model for brain amyloid deposition in an independent cohort was not validated. However, this is also a common limitation in previous studies. Hence, further large-scale, multicenter studies should be conducted. Second, we collected clinical data to define the presence or absence of dementia at baseline and not all participants with possible dementia could be excluded from the study. Our participants with MCI from the community had average annual conversion rate of 1.9% and reversion rate of 12.8%. Annual conversion rate from MCI to dementia in community-based studies was lower than that in clinic-based studies [80], whereas annual reversion rate from MCI to normal cognition in community-based studies was higher than that in clinic-based studies [81, 82]. The community sample had conversion rate of approximately 3% to 6% and reversion rate of approximately 25% to 30% [80, 81]. Therefore, the conversion and reversion rate in our cohort were relatively lower than those reported in previous community-based studies. Third, participants recruited from 855 community-dwelling individuals in this study were 122 with MCI or subjective memory complaints and the number of individuals with MCI who presented with abnormal PiB levels was relatively small. In addition, the sample size between the amyloid-positive (*n* = 68) and the amyloid-negative group (*n* = 214) was imbalanced, with a ratio of 1:3. This imbalance could lead to the construction of models with respect to the majority class. Therefore, more sampling techniques and larger cohorts are needed to address those sample size and class imbalance issues in future studies.

## Conclusion

In conclusion, we developed machine learning models for predicting PET amyloid positivity using easily available and noninvasive variables without the need to visit a hospital. Our models are useful for prescreening on enrollment of subjects who seems with brain amyloid deposition to reduce screen failure rate and trial costs in clinical trials. Furthermore, our models are useful for clinician to identify the individuals who really need to conduct lumbar puncture or amyloid PET scan even after the disease-modifying therapy getting approved.

## Data Availability

Data cannot be shared publicly due to ethical restrictions. The participants signed an informed consent form, which states that their data are exclusively available for research institutions in an anonymized form. The raw data used in this study contains sensitive and identifying information on individuals including gender, age, and education level that could compromise the privacy of research participants. However, the data that support the findings of this study are available upon ethical approval by the local ethics committee of the Oita University Hospital. Please contact the ethics committee of the Oita University Hospital. Email: rinrikenkyu@oita-u.ac.jp.
